# Supplementary material for: Detection of West Nile Virus Lineage 2 in Eastern Romania and First Identification of Sindbis Virus RNA in Mosquitoes Analyzed using High-Throughput Microfluidic Real-Time PCR
Source: Viruses. 2023 Jan 9;15(1):186. doi: 10.3390/v15010186 (PMC9860827; doi:10.3390/v15010186)
Supplement: Supplementary file 1 [file viruses-15-00186-s001.zip › viruses-2130008-supplementary.pdf]

Supplementary Table S1. Virus species targeted in high-throughput microfluidic real-time PCR and number of PCR designs per virus species, adapted from [26].

Virus number	Species	Number of PCR designs per virus	Family
1	Banna	1	*Reoviridae*
2	Banzi	1	*Flaviviridae*
3	Barmah Forest	1	*Togaviridae*
4	Batai	2	*Bunyaviridae*
5	Bwamba	1	*Bunyaviridae*
6	Chikungunya	5	*Togaviridae*
7	Dengue	8	*Flaviviridae*
8	Germiston	1	*Bunyaviridae*
9	Ilesha	1	*Bunyaviridae*
10	Inkoo	1	*Bunyaviridae*
11	Japanese encephalitis	7	*Flaviviridae*
12	Kedougou	1	*Flaviviridae*
13	Kokobera	6	*Flaviviridae*
14	Koutango	2	*Flaviviridae*
15	Middelburg	1	*Togaviridae*
16	Murray encephalitis	5	*Flaviviridae*
17	Ndumu	1	*Togaviridae*
18	Ngari	1	*Bunyaviridae*
19	Nyando	2	*Bunyaviridae*
20	O'nyong'nyong	1	*Togaviridae*
21	Orungo	1	*Reoviridae*
22	Pongola	1	*Bunyaviridae*
23	Rift Valley Fever	2	*Bunyaviridae*
24	Ross River	3	*Togaviridae*
25	Saboya	1	*Flaviviridae*
26	Semliki Forest	2	*Togaviridae*
27	Simbu	1	*Bunyaviridae*
28	Sindbis	6	*Togaviridae*
29	Spondweni	2	*Flaviviridae*
30	Tahyna	1	*Bunyaviridae*
31	Uganda	1	*Flaviviridae*
32	Usutu	1	*Flaviviridae*
33	Wesselbron	1	*Flaviviridae*
34	West Nile	8	*Flaviviridae*
35	Yaounde	1	*Flaviviridae*
36	Yellow fever	7	*Flaviviridae*
37	Zika	6	*Flaviviridae*
Total number of designs		94	

Supplementary Table S2. Comparison of the targets, the primers/probe sets used for WNV and SINV detection by High-throughput microfluidic real-time PCR amplification [26] and validation of the results by real-time PCRs screening for WNV [27] and SINV [29].

**High-throughput microfluidic real-time PCR amplification**				**Confirmation by real-time (WNV) or classical (SINV) PCRs**		
**Species**	**Primers (F and R) / Probe (P)**	**Sequence (5'-3')**	**Target**	**Primers (F and R) / Probe (P)**	**Sequence (5'-3')**	**Target**
WNV	WN_F	AAG TTG AGT AGA CGG TGC TGC	3'NTR	WNproC-F	CCTGTGTGAGCTGACAAACTTAGT	5′NTR/proC
	WN_R	AGA CGG TTC TGA GGG CTT AC		WNproC-R	GCGTTTTAGCATATTGACAGCC	
	WN_P	CGA CTC AAC CCC AGG AGG ACT GG		WNproC-P	CCTGGTTTCTTAGACATCGAGATCT	
	WN_1A_F	GTT GGC TCT CTT GGC GTT CT	C			
	WN_1A_R	GCA ATT CCG GTC TTT CCT CC				
	WN_1A_P	TCA GGT TCA CAG CAA TTG CTC CGA CC				
	WN_1B_F	GAA GTT AGC AGT CTA CGT TAG G	prM			
	WN_1B_P	TAT GGA AGA TGC ACC AAG ACA CGA CAC TC				
	WN_1B_R	GCA TAT CCA GGG TTT CTC AAG				
	WN_1C_F	TCA TGG TTG CGA CGT TCG TG	NS2a			
	WN_1C_P	AAG GCT AGG TGG ACG AAC CAG GAG AA				
	WN_1C_R	AAG TGT TGG TAA ACG TGA TGG C				
	WN_3_F	ATT TGA AGA ACC ACA TGC CAC G	E			
	WN_3_P	AAG CAA TCG GTG GTC GCC TTA GGT TCT				
	WN_3_R	TGC GCA TAC TCC ATA GGT CG				
	WN_4_F	GAT TGT GAA CCC AGG TCA GG	E			
	WN_4_P	CGT TGA TGT GGA CGC CTT CTA CGT GAT				
	WN_4_R	TGT TCC TCC AGT TCG TGT TTC				
	WN_2.1_F	GAG CTG TTT CTT AGC ACG AAG	C			
	WN_2.1_P	ATC TCG ATG TCT AAG AAA CCA GGA GGG C				
	WN_2.1_R	CAG ACT CAG CAT AGC CCT CT				
	WN_2.2_F	CAT GGA GAA AGT ACA CTG GCT A	prM			
	WN_2.2_P	ATA AGA AAG GAG CTT GGC TGG ACA GCA C				
	WN_2.2_R	GCA GTA GGA TAG CGA ACA CG				
SINV	SinFP	GGT TCC TAC CAC AGC GAC G	NSP1	A1-F (Forward)	5′-AAAGGATACTTTCTCCTCGC-3′	E2
	SinRP	TGA TAC TGG TGC TCG GAA AAC		A2-R (Reverse)	5′-TGGGCAACAGGGACCATGCA-3′	
	SinP	TTG GAC ATA GGC AGC GCA CCG GCT				
	Sindbis_I_F	GAA GGT AGA CGC CTA CGA AC	E1			
	Sindbis_I_R	ATG TAC TCT TGG TTG GTG GAA G				

High-throughput microfluidic real-time PCR amplification				Confirmation by real-time (WNV) or classical (SINV) PCRs		
Species	Primers (F and R) / Probe (P)	Sequence (5'-3')	Target	Primers (F and R) / Probe (P)	Sequence (5'-3')	Target
	Sindbis_I_P	ATG CGA CCA CTG TTC CAA ATG TGC CAC				
	Sindbis_2_3_F	GGT AGA CGC CTT CGA ACA TG	6k			
	Sindbis_2_3_R	GTG ACG TAC TCC AGG TTC GT				
	Sindbis_2_3_P	CGA CCA CTG TCC CAA ATG TGC CGA G				
	Sindbis_IV_F	AAT TCG AGG TAG TAG CAC AGC	NSP1			
	Sindbis_IV_R	GCA GAC GCA GTG ATA GTG GT				
	Sindbis_IV_P	AGG CCA CAC CAA ATG ACC ATG CTA ATG C				
	Sindbis_V_F	GTA GGA ATT AGG AAC ACT CTC G	NSP4			
	Sindbis_V_R	CTG ACT ATT TAG GAC CGC CG				
	Sindbis_V_P	CAG TTG CCG TAT CGA CCA GGT ACG A				
	Sindbis_VI_F	TAA CAG TGA AGA CGT GGT CAC	NSP2			
	Sindbis_VI_R	CTC CGT CTC TTG TTC CTT CAT A				
	Sindbis_VI_P	CGC TCT GGC CAG AAA GTT CGT CCG				

Supplementary Table S3. Mosquito species, collection site and Ct obtained in WNV microfluidic and confirmatory real-time PCRs.

Mosquito pool ID	Mosquito species	Collection site	County, Year	Viral target detected through High-throughput microfluidic real-time RT-PCR		Viral target detected for confirmation by Real-Time RT-PCR
				Ct mean, 3'NTR target	Ct mean, C target	Ct mean, 5'NTR-C target
28	*Culex pipiens* s.l	Natural site 2 (F)	Tulcea, 2019	26.7	27.5	32.6
129	*Culex pipiens* s.l	Natural site 1 (A)	Iași, 2019	22.5	ND	23.2
135	*Culex pipiens* s.l	Urban site 2 (D)	Iași, 2019	22.2	ND	20.9
136	*Culex pipiens* s.l	Natural site 1 (A)	Iași, 2018	22.5	ND	21.1
216	*Culex pipiens* s.l	Natural site 1 (A)	Iași, 2018	16.7	23.3	23.0
223	*Culex modestus*	Urban site 2 (D)	Iași, 2019	21.6	26.0	27.3
224	*Aedes vexans*	Natural site 2 (F)	Tulcea, 2019	20.0	26.6	26.4
257	*Culex pipiens* s.l	Natural site 1 (A)	Iași, 2018	18.3	22.9	25.0
316	*Culex pipiens* s.l	Natural site 2 (F)	Tulcea, 2019	24.5	ND	26.7
353	*Culex pipiens* s.l	Natural site 1 (A)	Iași, 2018	17.0	22.6	23.5

*ND = Not determined
